# Pancreatic Ductal Adenocarcinoma: Preclinical *in vitro* and *ex vivo* Models

**DOI:** 10.3389/fcell.2021.741162

**Published:** 2021-10-22

**Authors:** Beate Gündel, Xinyuan Liu, Matthias Löhr, Rainer Heuchel

**Affiliations:** ^1^Pancreas Cancer Research Lab, Department of Clinical Science, Intervention and Technology (CLINTEC), Karolinska Institutet, Huddinge, Sweden; ^2^Department of Upper GI, C1:77, Karolinska Comprehensive Cancer Center, Stockholm, Sweden

**Keywords:** pancreatic ductal adenocarcinoma, 3D cell culture, spheroid, reporter assays, organoids

## Abstract

Pancreatic ductal adenocarcinoma (PDAC) is one of the most overlooked cancers despite its dismal median survival time of 6 months. The biggest challenges in improving patient survival are late diagnosis due to lack of diagnostic markers, and limited treatment options due to almost complete therapy resistance. The past decades of research identified the dense stroma and the complex interplay/crosstalk between the cancer- and the different stromal cells as the main culprits for the slow progress in improving patient outcome. For better *ex vivo* simulation of this complex tumor microenvironment the models used in PDAC research likewise need to become more diverse. Depending on the focus of the investigation, several *in vitro* and *in vivo* models for PDAC have been established in the past years. Particularly, 3D cell culture such as spheroids and organoids have become more frequently used. This review aims to examine current PDAC *in vitro* models, their inherent limitations, and their successful implementations in research.

## Introduction

**FIGURE 1:**
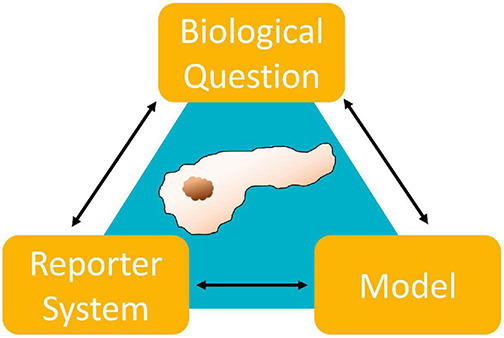
Illustrating the codependences of model, reporter system and biological question. A relevant model needs to be suitable to emulate the biological environment dictated by the research question. Additionally, there needs to be a reporter system which will allow the observation of explicit parameters suitable to answer that question. Finally, the model and reporter system need to compatible.

Over the past decades, researchers in cell biology recognized the limitations in clinical translation of both cell culture and animal models. Subsequently, effort was put into adjusting and accommodating to new demands. It is commonly accepted that there is no universally superior model but that instead the particular topic of research and the entailing experimental restrictions dictate model suitability (Figure 1). This review aims to survey common *in vitro* models used in pancreatic cancer research. As part of a review series, we will focus particularly on spheroid models, discussing examples of successful applications and limitations in PDAC research in this review.

Malignancies of the pancreas can originate from either the endocrine part or the duct system of the organ. The former are known as neuroendocrine tumors (NET) and have a much more favorable outcome than the latter. Pancreatic ductal adenocarcinoma (PDAC) as the most common tumor of the exocrine pancreas does often, but not exclusively, originate from the epithelial lining of the pancreatic duct. The classification of PDAC is done based on histological markers which overlap with those of healthy ductal epithelium. Of all the exocrine tumors, PDAC is by far the most common and accounts for the majority of deaths linked to pancreatic cancer. With a devastating median survival time of just 4.6 months (Carrato et al., 2015), PDAC was the 4^th^ leading cause for cancer related deaths in Europe in 2020 (European Union, 2021) and is projected to become the 2^nd^ leading cause of cancer related death by 2030 (Rahib et al., 2014).

Pancreatic ductal adenocarcinoma (PDAC) is characterized by dense and abundant desmoplastic stroma. The amount of cancer cells is often estimated at only 20% based on histological tissue inspection (Leppanen et al., 2019; Mayer et al., 2020). The most common cells in the tumor microenvironment (TME) are cancer associated fibroblasts (CAFs). They often stem from pancreatic stellate cells (PSCs) but can also arise from resident fibroblasts (Haber et al., 1999; Shek et al., 2002) and can be recruited from bone marrow derived stem cells (Marrache et al., 2008; Iwamoto et al., 2021). Upon injury to the tissue, caused by trauma or infection, these cells differentiate into myofibroblasts, which are rich in α-smooth muscle actin (α-SMA, ACTA2), and built up the major part of the extracellular matrix (ECM) (Haber et al., 1999). However, cancer cell lines also produce considerable amounts of matrix components (Löhr et al., 1994) and induce ECM formation in fibroblast by secretion of transforming growth factor β (TGF-β) (Abetamann et al., 1996; Löhr et al., 2001). Recent LC-MS/MS proteomics revealed that this property is not due to cell culture effects but verified the same findings in patient samples and a human-to-mouse orthotopic xenograft model (Tian et al., 2019). Furthermore, the type of secreted matrix components is not constant but changes as the cancer progresses. The main matrix components found in ECM are collagen types I and III, laminin, fibronectin, and hyaluronic acid (HA) (Fries et al., 1994; Löhr et al., 1994; Abetamann et al., 1996; Bachem et al., 2005; Naba et al., 2012). Like other glycosaminoglycans, HA is highly hygroscopic, causing a localized trapping of water which leads to increased interstitial fluid pressure and thus swelling of the tissue. This swelling exerts pressure, both onto the tumor itself and the surrounding healthy tissue, which is commonly summarized as solid stress (Nia et al., 2016; Voutouri et al., 2016). This compression triggers a number of mechano-sensitive responses, such as activation of latent TGF-β (Wipff et al., 2007), YAP (Zhang et al., 2014; Laklai et al., 2016), or fibronectin unfolding and interaction with collagen (Smith et al., 2007; Kubow et al., 2015). In a positive feedback loop TGF-β activates PSCs to differentiate into myofibroblasts which increase the contractile strain in the tissue (Biffi et al., 2019). Thus, solid stress causes a stiffening of the already dense matrix, exacerbating the restrictive nature of the TME in PDAC.

The physical changes of the TME have great ramifications for cancer therapy in PDAC. Commonly, blood vessels in solid tumors such as PDAC are leaky. Their formation is faulty due to the uncoordinated neo-angiogenesis found in tumors. This causes drugs of high molecular weight to exit the blood stream more easily in a tumor than in healthy tissue which leads to a drug enrichment in the target tissue (Maeda, 2012). This phenomenon is known as enhanced permeability and retention (EPR) effect and is widely exploited to reduce off-site effects in therapy (Maeda et al., 2000; Maeda, 2012). The EPR effect, however, is compromised in PDAC. Tumor blood vessels collapse due to their flawed construction and the pressure induced by the stroma and blood flow is more strongly directed out of the tumor rather than into it (Maeda, 2012). Unlike many other cancers, neo-angiogenesis is not common in PDAC either and the resulting lack of blood flow creates a highly hypoxic TME.

Hypoxia in tumors has long been linked to increased metastatic potential and, consequently, poor patient outcome in PDAC and other cancer types (Brizel et al., 1996; Höckel et al., 1996; Chang et al., 2011). Hypoxia represents evolutionary pressure for cancer cells. The ones which manage to adapt, are more resilient toward poor metabolic conditions as a result. Additionally, hypoxia itself sharply reduces the effectivity of radiotherapy as it relies on generating reactive oxygen species from elemental oxygen.

Unsurprisingly, these circumstances have made treatment difficult. Where applicable, surgery remains the best option for survival for now. However, improving *in vitro* models harbors two major advancements to improve patient outcome: the development of *in vitro* models describing the tumor microenvironment more accurately and thus furthering drug discovery; and by facilitating personalized medicine in the form of patient derived organoids (PDOs) or patient derived xenografts (PDXs). Combined, as *in vitro* models increasingly reflect the complexity of PDAC more accurately, they represent a better chance to identify ways to overcome resistance to conventional treatments.

## 2D Cell Culture

2D cell culture has been the standard of operating procedure for molecular life sciences for good reasons. It is easy to control and manipulate in experiments and hence provided good understanding for the fundamental processes in living cells. Compared to cell-free systems, e.g., in drug design screens, the introduction of cellular systems introduced parameters like membrane permeability, the impact of naturally occurring agonists and antagonists on the intended target and of course the cytotoxicity of the tested compounds (Blay et al., 2020). The technical benefit of using 2D cell culture was indeed so great that commercial assays are often designed based on 2D cell culture, also given how widespread and easily accessible this model is.

This simplicity, however, is not sufficient when investigating increasingly complex systems such as cancer. Understanding the microenvironment of a tumor has been recognized as essential in eventually overcoming the challenges and heterogeneity of cancer. Pancreatic cancer in particular has a highly altered microenvironment (Kleeff et al., 2007), marked by excessive desmoplasia, hypoxia and poor nutrition. 2D cell culture fails to simulate this environment sufficiently on several accounts. Most strikingly, cells grow in monoculture unlike tissue, which consists of a multitude of different cell types. The surroundings of cells are also severely altered in 2D cell culture where there is none of the extracellular matrix preserved or commonly replicated. An inevitable drawback shared by any *in vitro* system is the selection for fast growing cells. In contrast, healthy tissue grows slowly and tightly regulated, even after injury. Additionally, while incubation chambers are supplied with carbon dioxide, they rely on normoxia when it comes to the oxygen content in the medium.

However, there are ways which enhance the biological relevance of this cell culture system. A more hypoxic environment can be achieved by using hypoxia chambers which are commercially available (Abdalla et al., 2019). With these, the cells can be cultivated under an altered atmosphere with partial gas pressures that resembles the ones found in tissue more closely (Geismann and Arlt, 2020). Another example is the use of trans-well plates which allows the cultivation of different cell types in one well. The medium containing signaling molecules and metabolites can diffuse through a membrane enabling crosstalk of solvent molecules. However, cell-cell-contact and its subsequent signaling is not possible.

Conclusively, PDAC research has recognized the complexity and significance of the TME with high levels of dense ECM. More detailed investigations into PDAC consequently require models which include these properties.

## 3D Cell Culture

When it comes to 3D cell culture, there are several levels of model complexity and consequently biological relevance. What they all have in common is that cells are not cultivated as a monolayer. By various means, which will be discussed in detail below, some aspects of the three-dimensionality of tissue are simulated or even preserved. Another commonality is that these models are not as established yet despite simulating *in vivo* conditions better compared to 2D cell culture models. For example, failure of potential drug candidates at early stages of drug development in more advanced models might prevent costly failures at later stages.

However, more advanced models also have drawbacks. More complex models are inherently less consistent as simpler models. Consequently, more complex models also generate less consistent samples. This reduced model fidelity carries the risk of masking significant results. It is hence an ongoing struggle to minimize this sample background heterogeneity by streamlining established protocols.

### Spheroids

Spheroids are solid cell clusters generated from established 2D cell lines and do not require many additional changes in culture conditions compared to the 2D requirements. There are different types of spheroids and several ways to generate spheroids and based on the method used, the properties of the spheroids will differ (Table 1). Consequently, it is important to keep the methodology in mind as a source of heterogeneity when comparing different findings, especially when the conclusions drawn from the experiments contradict one another.

**TABLE 1:** Comparison of different techniques used for spheroid formation and their attributes (Merck KGaA, 2021; The Cell Culture Dish, 2021).

**Attributes**	**Matrix-embedded**		**Hanging drop**	**ULA-plate**	**ULA-plate with crowding agent**	**Bioreactor**	**Microfluidic system**
	** Animal derived matrices**	**Synthetic hydrogels**					
Consistency of samples	+	++	+++	++	++	−	++
Cost efficiency	−	−	+	+++	+++	−	−
High throughput	+	+	++	++	++	+++	−
Long term culture	++	++	+	++	+	+++	+++
Sample retrieval	+	+	++	+++	+++	+++	−
Image analysis	+	+	+	++	++	−	+++

*Suitability: +++ = High; ++ = Medium; + = Low; − = Very low. ULA = ultra low attachment.*

Common to all 3D culture is that the cells are forced not to adhere to the plastic surfaces of the culture vessels, but instead aggregate with other cells. To this end ultra-low attachment plastics as well as less costly methods using simple non-adherent plates in combination with crowding agents like methylcellulose or agarose coated plastics have been developed (Carlsson and Yuhas, 1984; Longati et al., 2013). Spheroids can be either grown just in liquid (media), embedded in or on matrix or by using a microfluidic platform.

The most common matrices used for spheroid generation are Matrigel and collagen hydrogels. These two natural matrices supply common ECM proteins, such as collagens and fibronectin, which allow cell-cell and cell-matrix interactions, e.g., by cadherins and integrins. A more specialized matrix is HA-based and for PDAC spheroids of particular interest as HA is a main component of the TME and the major driving force for the tumor interstitial fluid pressure (Scaife et al., 2008; Liu et al., 2018). Tissue stiffness was determined by atomic force microscope (AFM) to increase from 0.4 kPa in normal pancreas tissue to 1.2 kPa of a PDAC tumor (Rice et al., 2017). Collagen matrices have a compression force more akin to healthy tissue while modified HA matrices reach compression forces up to 1.5 kPa which compares well to the pressure measured in PDAC tumors (Chang and Lin, 2021). A major drawback of using natural compounds is their batch-to-batch variability. The substitution with synthetic polymers alleviates this problem and introduces more control and consistency to the model. The most widely used synthetic polymer is polyethylene glycol (PEG), a substance widely used in biomedical context due to its non-toxicity and non-immunogenicity. Polyethylene glycol can be modified to show properties similar to ECM proteins and enable cell aggregation (Liu and Vunjak-Novakovic, 2016).

While organoid culture, described below, relies heavily on these matrices, spheroid culture has alternative options by using microfluidic systems. The simplest way is preventing cell adhesion by using coatings, such as agarose (Carlsson and Yuhas, 1984; Ma et al., 2012). This approach is quite easy to accomplish and inexpensive but lacks spheroid uniformity, a feature important for consistent results. The same problem arises when using bioreactors, such as the spinner flask. While this method has a very high yield of spheroids and enables ongoing growth and culture, the produced spheroids come in any shape which limits their experimental use (Cui et al., 2017). As reproducibility and uniformity of size and shape is very important in research, the hanging drop method was favored over the previously mentioned methods (Timmins and Nielsen, 2007). Although more labor intensive than other spheroid generation methods and smaller overall size of spheroids, it provided more consistency and allowed medium throughput analyses (Cui et al., 2017). A recent advancement was combining aspects of microfluidics and matrix assisted growth by supplying the growth medium with the crowding agent methylcellulose (MC) in combination with non-adherent cell culture vessels (Longati et al., 2013). Like solid matrices, MC increases the viscosity of the medium enough to prevent cell sedimentation but unlike natural polymers it is not subject to batch-to-batch-variation. Spheroids are grown one per well, in scale to the well size, so it does not reach the high throughput levels of bioreactors. However, with liquid handling equipment commonly available at high throughput facilities spheroids were successfully grown even in 1536 well plates (Madoux et al., 2017). It grows consistently sized spheroids which are considerably larger than those produced by the hanging drop method, creating more distinct internal gradients.

Even the most basic form of spheroid culture which only uses a single cell type improves on mirroring the conditions of tissue compared to 2D cell culture (Friedrich et al., 2009; Longati et al., 2013; Wagner et al., 2013). By shielding the core of the spheroid from the medium, gradients of oxygen, nutrients, metabolic waste products and signaling molecules are generated. Often the core of the spheroid shows necrosis upon prolonged culture while the exterior layers still proliferate. As such spheroids might be viewed as avascular minitumors.

Dense stroma is a key hallmark of PDAC. Spheroids were shown to build up some matrix components (Longati et al., 2013), however, fall short in mimicking the combined physical properties of the tumor, like stiffness and compression, and thus cannot inherently model solid stress. External induction of solid stress can be simulated using a collagen-1-matrix for embedding.

Spheroids can be grown in coculture in several ways to investigate cell-cell-communication. The before-mentioned trans-well method can be used, e.g., a spheroid in solution and a 2D cell culture of CAFs, or a solid spheroid with immune cells in solution (Kuen et al., 2017). Even a combination of 3 types of cells was reported with heterospheroids of different pancreatic cancer cell lines and CAFs being exposed to monocytes. Histology allowed then to assess infiltration and drug response (Kuen et al., 2017).

Additionally, spheroids can also be generated from more than one cell type forming heterospheroids (Norberg et al., 2020). Subsequent analysis of crosstalk between the different cell types requires pre-labeling of these. For example, cell lines expressing fluorophores are often used to distinguish cell types within the spheroid or during cell sorting or image analysis (Öhlund et al., 2017). Cell sorting comes with the drawback of having to generate single cells from spheroids which entails substantial stress on the cells and sample loss. Other pre-labeling techniques like the use of isobaric tags have been carried out to investigate the proteomic shift between 2D and 3D culture in PDAC (Samonig et al., 2020) and to show how drug-induced Akt-inhibition increases the stemness of pancreatic cancer spheroids (Arasanz et al., 2020).

Alternative to pre-labeling, different cell types can also be vitrually “sorted” after cultivation by using cells from different species and exploiting the small differences in species specific DNA/RNA sequences (Conway et al., 2012; Liu et al., 2021). Our lab established one such model by generating heterospecies heterospheroids using human Panc1 cells and immortalized mouse pancreatic stellate cells (imPSCs) (Norberg et al., 2020; Liu et al., 2021). This allowed for the analysis of cell-cell crosstalk and could show that the coculture in heterospheroids substantially changed the gene expression pattern in key cancer pathways, PDAC type stratification as well as PSC/CAF phenotype (Liu et al., 2021). The RNA profile of Panc-1 in heterospheroids was more reflecting squamous like phenotype compared to Panc-1 in monospheroids. The CAF phenotype was shifting from myCAF to iCAF due to the presence of Panc-1 cells in the heterospheroids.

It could also be repeatedly shown that the spatial distribution of cells and the resulting gradients have substantial effects on chemosensitivity. Especially in the context of pancreatic cancer research with a large focus on drug discovery, these findings add weight to the progression toward three-dimensional cell culture as the base line for medical research which aims to identify novel treatment options.

As spheroids are grown from 2D cell culture sources, some of the limitations carry over while some characteristics are improved upon. Cell line identity can drift the longer a cell line is kept in culture. Especially cancer cells, which have lost many DNA control/repair mechanisms, are more prone to accumulate mutations. In order to limit the influence of loss of cell line identity, cell lines should be tested regularly by short tandem repeat analysis and discontinued after 40-50 passages (Reid et al., 2013). Another limitation is that also spheroids cannot model the heterogeneity of PDAC. For one, established pancreatic cancer cell lines are limited in number (Moore et al., 2001) and do not reflect the genetic heterogeneity of mutations found in patients. Nor do co-cultured cell lines such as PSCs as recently shown (Lenggenhager et al., 2019). An additional problem lies in that not all cancer cell lines form spheroids and even less form heterospheroids with other cell types. Consequently, any findings carried out using only one model are not guaranteed to apply to all cell lines, all models and ultimately not all PDAC patients. PDAC, like many other cancers, is inherently heterogeneous and has several sub types. In this context, different 2D cell lines grown as spheroids can be viewed as models for different subpopulations of patients.

Other methods rely on single cell sedimentation into cell clusters (Lee et al., 2019). Uniform indentations in a silicon matrix allow for the sedimentation of 2D cells which then pack densely as a result of gravity. This is one of the novel approaches to develop 3D cell culture further, but it is too early to be assessed/discussed in a greater context for now.

#### Reporter Assays for Spheroid Cultures

Modern research in the field of cell biology is mostly dependent on reporters that rely on optical permeability at one point of the analysis with only few exceptions, e.g., radioactive tracers. We then interpret these optical readings in a biological/physiological context to draw conclusions. As such, models heavily rely on allowing light transmission.

Methods which treat spheroids as small tissue pieces were designed to deal with samples which cannot readily pass light. Consequently, practices like paraffin or OCT (optimal cutting temperature) compound embedding for immuno-histochemistry (Maftouh et al., 2014) following sectioning the sample are readily compatible with spheroid research and are being carried out routinely.

Commercially available biochemical assays have simplified sample analysis substantially by standardizing and simplifying protocols hence making experiments more reproducible between different research groups. However, most of these assays were designed for 2D cell culture which readily fulfills the important prerequisite of allowing light passage. Spheroids on the other hand exhibit a compact structure which allows for very little light transmission. Consequently, adapting reporter systems to be used for spheroid research is not trivial.

There are some classes of reporter assays which commonly adapt well to spheroid culture: those that involve lysis of the spheroid and those that test only a reporter in the culture medium. As mentioned previously, spheroid culture can be carried out without using a solid matrix but an altered medium instead. The combination of a liquid medium-based spheroid culture with colorimetric or fluorescent reporters which only samples the medium even allows a set up for high throughput screens.

More detailed analyses of the medium are being carried out as well. Metabolomics, i.e., detection of radioactively labeled substrates in combination with NMR have been successfully applied to Panc-1 spheroids, or more precisely to the medium they were cultivated in Fan et al. (2018).

An example for a commercial adaptation to 3D samples is the cell viability kit CellTiterGlo 3D^TM^ with enhanced lytic capabilities. As a result this assay is widely and successfully used (Norberg et al., 2020; Liu et al., 2021), and is the golden standard for cell viability in high throughput screens using spheroids. The APH assay is another cell viability assay optimized for 3D cell culture (Longati et al., 2013). It measures the activity of acid phosphatase in the cells. However, this assay requires a high pH step which impedes its implementation in high throughput settings due to its corrosive effects on metal parts of liquid handling equipment.

**FIGURE 2:**
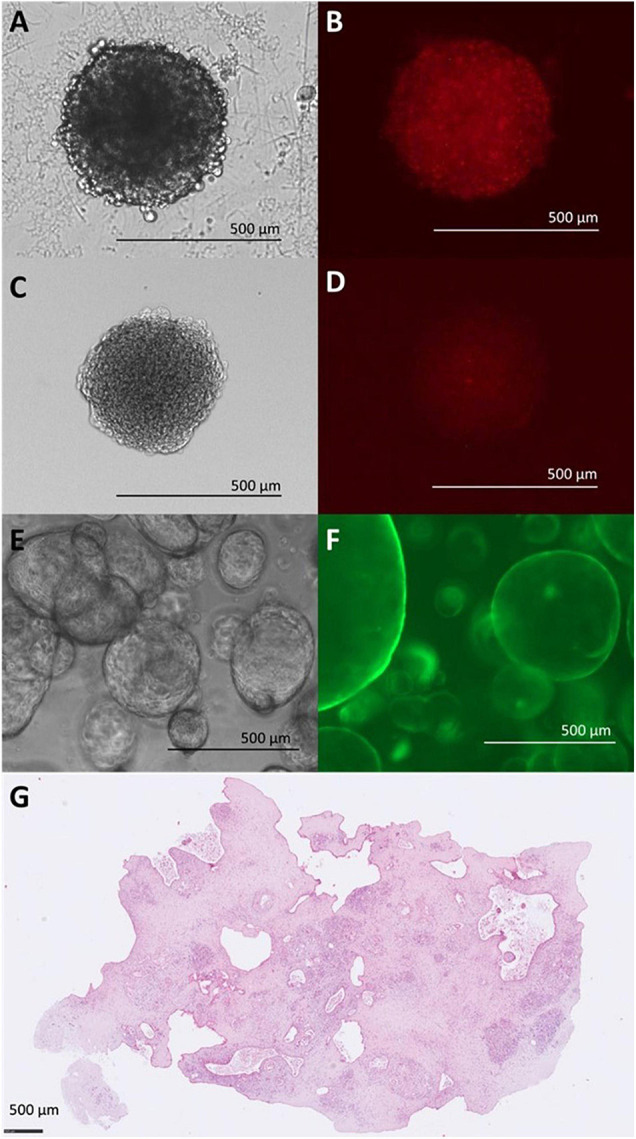
Limitations of light microscopy for different *in vitro* models. **(A)** Bright field (BF) image of Panc-1 and imPSC heterospheroid at 4d. **(B)** Detection of mCherry fluorophore in Panc-1 and imPSC heterospheroid at 4d. Evenly distributed fluorescent signaling despite unevenly distributed cells in a spheroid. **(C)** BF image of Panc-1 and imPSC heterospheroid at 4d after clearing procedure. Clearing involved dehydration with ascending concentrations of ethanol causing some shrinking of the spheroid. **(D)** Detection of mCherry fluorescent protein in Panc-1 and imPSC heterospheroid at 4d after clearing. Fluorescent signal diminished due to clearing procedure but distribution of the signal aligns with cellular distribution with more concentrated signal in the center. **(E)** BF image of hollow organoids derived from a mouse tumor at 2d. **(F)** Detection of eGFP fluorophore in hollow organoids derived from a mouse tumor at 3d. The transparency of the organoid line allows unhindered observation. **(G)** Organotypic slice culture image after 72h in *ex vivo* culture. H&E staining. Images **(E,F)** were kindly provided by Daniel Öhlund, Umeå University, Sweden. Image **(G)** was kindly provided by Carlos Fernández Moro, Karolinska Institutet, Stockholm, Sweden.

While these options offer a wide field of research, the most common reporter remains elusive: fluorescence. We previously mentioned fluorescence as a simple read out to distinguish different cell types in live cell imaging by making use of continuously expressed fluorescent proteins, but many available assays rely on activatable fluorescent probes as well. However, the larger the spheroid, the less applicable fluorescence as a reporter becomes. The sheer density of the “tissue” prevents light passage (Figures 2A,B). Confocal microscopy can be used to observe tissues too dense for brightfield imaging. However, the more elaborate image capture then limits the throughput capacity. Tissue clearing alleviates this problem altogether but also relies on embedding the tissue and precludes further culturing (Figure 2C). Spheroid protocols which provide highly homogenous spheroids can overcome this difficulty with larger sample number, so that different spheroids serve as single data points in timelines rather than one spheroid being continuously observed. A common problem with clearing protocols is the diminishing of pre-labeling fluorophores (Figure 2D). Fluorophores used on already cleared samples, e.g., during immunohistochemical staining, are not affected.

Single cell-analysis like flow cytometry relies on creating single cells from a spheroid which often entails substantial loss of cells. Additionally, there is uncertainty about the homogeneity of single cell origin. Central parts of the spheroid may not be fully separated into single cells and gated out of the analysis as a result. This would then bias the analysis toward cells on the outside of the spheroid which were less subjected to existing gradients. In the case different cell types are mixed in spheroids, the methods used to prepare single cell suspensions might preferentially harm one cell type introducing a different type of bias.

An ongoing trend in research is to add a spatial parameter to any given optical output and create chemical images of samples. One such method is matrix assisted laser desorption ionization mass spectrometry imaging (MALDI-MSI). The spatially targeted MS-analysis allows for example to determine drug penetration in spheroids (Mittal et al., 2019).

To our knowledge spatial transcriptomics has not yet been introduced in PDAC spheroid research. This method, however, would be interesting to use in order to investigate the RNA profile along the gradients that build up in a spheroid. While single cell RNAseq (Liu et al., 2021) and transcriptomic profiling (Yang et al., 2018) have already provided much information about gene expression in pancreatic cancer spheroids, succeeding in adding the spatial aspect to this data would offer even more insight. High definition spatial transcriptomics (HDST) can be carried out with a resolution of 2 μm (Vickovic et al., 2019). Given the diameter of spheroids varying between 200 and 600 μm in diameter, based on cultivation method, spatial transcriptomics would provide a differentiated view on the impact of metabolic gradients on RNA expression.

Fourier-transform infrared (FTIR) spectroscopy imaging was successfully used for detecting necrosis in melanoma spheroids, a useful tool when determining toxicity of compounds in research (Srisongkram et al., 2020). The advantage of this method over others is the multivariate analysis that it is commonly linked with. It can quantify RNA, lipids and DNA and even subclasses of proteins present, as well as their folding status. As such it is a more comprehensive analysis of fewer samples. This method has not been carried out with PDAC spheroids yet but could provide interesting insights, for example when trying to determine which changes occur to the ECM during different treatments. This may reveal insights as to why certain drugs fail in 3D which seemed promising in 2D as FTIR imaging was also able to find substantial differences in biochemical composition between the two models (Srisongkram et al., 2020). Additionally, FTIR imaging can also be taught to identify certain compounds similarly to MALDI-MSI by incorporating specialized spectral libraries and so could also serve to keep track of drug delivery and metabolization.

However, what image-based analyses of spheroids have in common is that they are not adaptable to high throughput yet and so cannot replace 2D-cell-culture oriented assays. In the future, with implementation of machine learning, improvements in image acquisition and a more automated image analysis, image-based analysis has the potential to enhance and replace some of the methods for spheroid research described here. In the meantime, more work needs to be invested into establishing robust and efficient reporting systems as spheroids represent a significant improvement compared to 2D cell culture.

Particularly methods which are high-throughput ready need to be established on a broader base. The main parameter currently is cell viability but in order to distinguish PDAC-specific toxicity from general cell toxicity more information must be acquired in large drug screens, as for instance metabolic parameters such as pH, lactate or glutamate accumulation and consumption of glucose and glutamine.

### Organoids

Since this review is part of a review series, we will not focus as much on organoids as this topic is being covered in several entries of the series.

In comparison, organoids are 3D structures that mimic properties and tissue organization of the organ the cells are derived from. An important aspect of organoids is the self-assembly into a micro tissue. As such their structure is more complex than that of spheroids and 2D cell culture while still being grown under laboratory conditions. While spheroids are grown from established 2D cell lines, organoids also known as PDOs are cultivated using primary cells. Consequently, they represent the heterogeneity of cancer much more accurately than spheroids or 2D cell lines. Organoids often preserve the polarity and gene expression pattern of the original tumor during early passages (Baker et al., 2016). Organoids can grow as hollow spheres (Figure 2E) or small, more solid spheres which allow light passage and the use of fluorescence for analysis (Figure 2F). All parameters considered, organoids seem like a better suited candidate for *in vitro* drug testing.

However, the requirements for successful culture are considerably higher and more expensive than that of spheroids. Spheroids are easily propagated, quickly grown to the required amount and inexpensive to maintain. On the other hand, the self-organization of organoids into a tissue relies on signaling, both via solvent molecules, cell-matrix- and cell-cell-interactions. The most widely used matrix to facilitate this signaling is Matrigel^®^. It is animal-cell derived and provides a complex mix of matrix components that is able to mimic the natural matrix composition of the basement membrane. However, also due to its animal origin it is subject to significant batch-to-batch variations which negatively affect the reproducibility of organoid research. In addition, Matrigel is rather soft compared to the stiff collagen-I-rich stroma characteristic for PDAC (Chang and Lin, 2021). Attempts to move into a more homogenous matrix have not been successful yet but remain a target of intensive research. This obligatory embedding in matrix also poses a problem for sample accessibility. Whereas spheroids grown in liquid medium can be collected and split with ease, organoids need to be separated from the matrix. Precise organoid sample aliquots are not possible without breaking them down to single cell level, a condition some organoid lines have problems to recover from.

Given these difficulties, organoids have not yet firmly established their implementation into high throughput drug screening. However, with further improvements of the model, organoids will also play an increasingly important part in the 3Rs of humane animal research, to replace, refine and reduce animal experimentation in modern research as well as become a platform for drug screens of varying scale.

The most striking benefit of organoid research are the possibilities for clinical application. As previously mentioned, PDOs are generated from patient tumor tissue. Hence these organoid lines closely resemble the *status quo* of an individual patient. Potential therapies can then be tested *in vitro* for effectiveness before being administered to the patient, avoiding unnecessary side effects from ineffective treatments (Huang et al., 2015, 2020; Tiriac et al., 2018a). However, as before mentioned the median survival time for PDAC is around 6 months, meaning that a significant number of PDAC patients have a shorter life expectance than it takes to generate enough organoid material and perform the drug testing. To make a difference for patient therapy, organoid establishment, drug screen and data analysis must be carried out within a time frame short enough to warrant delaying immediate treatment. Current approaches cannot supply this information quickly enough yet. With better diagnostic markers being investigated in parallel by many research groups, this strained schedule could become more relaxed in the future. Until then, only the roughly 25 percent of patients amenable to surgery and thus with longer life expectancy can take advantage of this approach. Proposals to incorporate small scale PDO screens into clinical practice have been made as well (Frappart et al., 2020). In very recent years, protocols to derive organoids from fine needle aspiration biopsies are being established which could provide the necessary drug testing platform for patients not suitable for tumor resection (Tiriac et al., 2018b). However, acquiring enough cancer cells from the sampled tissue currently necessary for organoid establishment presents a major hurdle, especially when using fine needle biopsies in PDAC.

### Organotypic Slice Culture

This model uses precision cut slices of tissue which is cultured submerged in medium adjusted to the tissue used (Moro, 2021). Only recently has this model been used in PDAC research (Jiang et al., 2017; Misra et al., 2019; Moro, 2021).

An outstanding benefit of this type of *in vitro* model is the maintaining of the TME and the spatial information of the tumor (Figure 2G) in combination with time resolved analysis. The activation and effects of CD8^+^ T-cells following treatment was demonstrated using live-cell imaging in combination with confocal microscopy (Seo et al., 2019). Additionally to the tissue, the supernatant can be analyzed as well, e.g., to detect soluble signaling molecules (Jiang et al., 2017; Seo et al., 2019).

Organotypic slice culture can be viewed as an alternative to organoid culture in personalized medicine. Like organoids, it maintains the genetic tumor heterogeneity of the patient’s tumor. Additionally, it retains the stromal environment, which gets inevitably lost during organoid generation. In detail, it could be shown that proliferation rate and grade of tumor differentiation could be maintained throughout culture duration of 4 days (Misra et al., 2019). Tissue slices also responded in a dose and time dependent manner to drug treatment which was confirmed by immunohistochemical measurements of cellularity and cleaved caspase-3 positive cells (Misra et al., 2019). A benefit compared to organoid culture is its immediate availability.

We discussed earlier the time sensitivity of PDAC treatment and how organoid culture takes too long under current conditions to be a tool in personalized medicine. Organotypic tissue slices, on the other hand, are available quickly and can be used to screen for specifically effective anti-cancer drugs (Ghaderi et al., 2020). Naturally, the size of the tumor limits the number of samples and the number of drugs which can be tested. Consequently, organotypic slice culture cannot serve as a model for drug discovery but remains an interesting tool for the advancement and clinical translation of personalized medicine in PDAC.

Conclusively, 3D cell culture is a diverse field of ongoing research with significant improvements compared to 2D cell culture in modeling a complex disease like PDAC (Table 2). However, more reporter systems which are tailored to these models are still required to make them an even more advantageous part of PDAC research.

**TABLE 2:** Comparison of relevant parameters of spheroid, organoid and organotypic slice model.

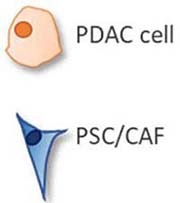	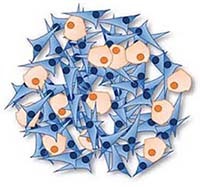	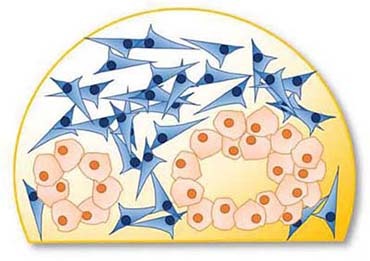	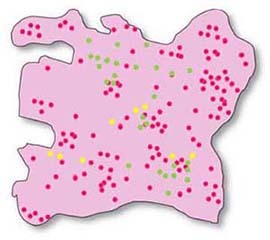
	**Spheroid model**	**Organoid model**	**Organotypic slice culture**
Derived of	established 2D cell lines	primary tissue	Primary tissue
Complexity	homogenous sample generation with consistent growth progression cannot model tumor heterogeneity	varied organoid growth density and limited growth predictability retains the genetic expression pattern of the original tumor	cultivation of precision-cut tumor slices retains TME, its spatial distribution and tumor differentiation/grade
Co-culture system	• heterospheroid formation limited to few cell lines • trans-well co-culture with monospheroids • differentiated cell-type-specific analysis of crosstalk possible	• co-culture inside Matrigel dome • co-culture with suspended cells	• contains all cells of the patient’s TME • additional non-adhesive cells can be added to medium to observe infiltration
Availability	compatible with regular cell laboratory facilities	requires additional storage, management and cultivation resources	compatible with regular cell laboratory facilities
Costs	similar to 2D culture, depends on protocol	additional costs caused by Matrigel and medium supplements	additional medium components necessary
Most applicable reporter systems	+ analyses of the medium + imaging after embedding + analyses of lysates use of radioactive tracers	+ image-based analysis with or without embedding + analysis of lysates + use of radioactive tracers	+ immunohistochemistry + live-cell imaging with confocal microscopy + medium analysis
Complications for reporter systems	limited light transmission of whole spheroids	limited accessibility due to matrix embedding and matrix interactions	tissue architecture prevents cell-type-specific biochemical analyses
High throughput application	analysis of medium and spheroid lysis	AI-assisted image analysis	Not applicable to high throughput due to very small sample size

## Spheroids as a Steppingstone

Spheroids are not exclusively used as a model for research. Instead, they are also used as a tool to refine animal models and to advance other *in vitro* models. This last part of the review will focus on the implementation of spheroids in other preclinical models.

### Xenografts

Xenografts are an *in vivo* model commonly used in translational research. Typically, suspended cells are injected orthotopically or subcutaneously. However, when injecting spheroids instead of 2D cells the resulting tumors grew more homogenously as well as more successfully. As such the implementation of spheroids reduced the number of animals subjected to cell injection as well as refining the *in vivo* model (Valta et al., 2016; Durymanov et al., 2019; Huang et al., 2020).

For PDAC there was one more substantial improvement. When transplanting spheroids consisting of Panc1 cancer cells and 3T3 fibroblasts, the resulting tumors contained more stroma than when suspended cells were injected, hence creating tumors which also more accurately resemble a patient’s tumor (Durymanov et al., 2019).

### Organ on Chip

Organ on chip systems use cells suspended in hydrogels in a small glass chamber. This model specializes on mimicking the influence of tissue-tissue interfaces or fluid-tissue interfaces. The latter is achieved by using microhydraulic systems which simulate blood circulation. The central drawback of this model is the inability to collect cells for sampling so any results need to be image-based (Tomás-Bort et al., 2020).

Models including spheroids grown from PDAC lines were established on several accounts. Established PDAC cell lines were embedded in collagen and offered ongoing nutritional perfusion (Beer et al., 2017). This model proved highly resistant to cisplatin and identified the matrix-interaction as a crucial factor in model establishing. Despite not forming spheroids, the cells responded more akin to those in spheroids rather than those in ECM-free 2D culture (Beer et al., 2017).

An organ on chip model was also designed to investigate solid stress found in PDAC. By increasing the interstitial fluid pressure to match that of a patient tumor an upregulation of the multidrug resistance protein family could be observed (Kramer et al., 2019).

A microfluidic system of Panc-1 and PSC cells was established to demonstrate the promotive effect of PSCs on cell motility (Lee et al., 2018). Additionally, an increased gemcitabine resistance facilitated by PSCs was demonstrated. The addition of medium circulation represents a platform to not only test for drug responses but also to test for dosage and treatment schedules.

A relatively new application is investigating multi-organ crosstalk (Wagner et al., 2013; Schimek et al., 2020). There, a liver spheroid was introduced to metabolize administered drugs to observe any possible adverse effect not only of the original drug but also its metabolized products.

While not yet attempted to our knowledge, the combination of liver metabolism to PDAC drug trials would represent a considerable advancement. Not only due to the added metabolic degradation of the drugs but also to investigate liver toxicity. As metastases of PDAC are commonly observed in the liver, this organ is of additional interest in research seeking to improve PDAC treatment.

### 3D Bioprinting

Bioprinting uses cells typically suspended in hydrogels or other solidifying scaffolds to precisely determine the distribution of different cell types to one another. Unlike previously mentioned methods it thus seeks to recapitulate the morphology of organs or organ systems.

The use of scaffold also serves the purpose to give cells the correct cues for migration and differentiation which are naturally provided by the ECM.

Another novel approach was using scaffold embedded spheroids instead of scaffold embedded cells (Goulart et al., 2019). Compared to single cells, hepatic spheroids showed a more balanced metabolism and more importantly preserved the cell identity of the hepatocytes even in prolonged culture.

The bioprinting of neural spheroids was also recognized as more advantageous compared to single cell printing. Again a prolonged longevity of the culture could be observed, based on the enhanced self-renewal (Han and Hsu, 2017).

The importance of modeling the appropriate ECM for PDAC was also recognized in the area of bioprinting. However, only few studies have so far been carried out with PDAC cells. PDX derived cells were embedded along PSCs and human umbilical vein endothelial cells (HUVECs) (Langer et al., 2019) which created a dense and active stroma over time.

To summarize, spheroids seem to represent a way to stabilize the cellular identity. This conservation is especially important for fields of bioprinting which are slowly progressing into clinical application. Additionally, however, the benefit of *in vitro* models aiding in improving upon established models like the murine xenograft should not be overlooked.

## Conclusion

To model complex and heterogenous pathologies like cancer, *in vitro* models must move beyond 2D cell culture as a failure-rich history in PDAC research has demonstrated. Likewise, the methods by which we build, interrogate and interpret these models must keep pace and develop further to meet the changing and increasingly complex questions of frontline research. Spheroids in particular exhibit great balance in recapitulating tissue conditions more authentically while also allowing controllable conditions which can be easily manipulated in experiments. Alongside spheroids, all 3D cell culture models will further expand our understanding how the TME can be modified in order to improve patient treatment. High throughput drug screenings and personalized medicine are merely the most prominent examples how 3D cell culture models could translate into relevant preclinical applications; the clinical effectiveness and truth of which will only be revealed by time.

## Author Contributions

All authors contributed to collecting literature, and to writing and revising the review.

## Conflict of Interest

The authors declare that the research was conducted in the absence of any commercial or financial relationships that could be construed as a potential conflict of interest.

## Publisher’s Note

All claims expressed in this article are solely those of the authors and do not necessarily represent those of their affiliated organizations, or those of the publisher, the editors and the reviewers. Any product that may be evaluated in this article, or claim that may be made by its manufacturer, is not guaranteed or endorsed by the publisher.
